# Comparison Study of Perceived Neighborhood-Built Environment and Elderly Leisure-Time Physical Activity between Hangzhou and Wenzhou, China

**DOI:** 10.3390/ijerph17249284

**Published:** 2020-12-11

**Authors:** Jiabin Yu, Chen Yang, Shen Zhang, Diankai Zhai, Jianshe Li

**Affiliations:** 1 Faculty of Sport Science, Research Academy of Grand Health, Ningbo University, Ningbo 315211, China; nbuzhangshen@outlook.com (S.Z.); zdk5780245@outlook.com (D.Z.); lijianshe@nbu.edu.cn (J.L.); 2 Department of Kinesiology and Physical Education, McGill University, Montreal, QC H2W 1S4, Canada; chen.yang4@mail.mcgill.ca

**Keywords:** built environment elements, aging over 60 years, recreational physical activity, empirical study

## Abstract

Physical activity and health are of significant importance for the rapid aging population in China. Built environment has been suggested to be associated with elderly physical activity and health. However, the association differences between cities remain unclear. Perceived built environment scores and elderly leisure-time physical activity (LTPA) of 308 elderly in Hangzhou and 304 elderly in Wenzhou were collected using Neighborhood Environment Walkability Scale and International Physical Activity Questionnaire, respectively. A multivariate linear regression method and T-test were used to analyze of the associations between elderly LTPA and built environment and the differences between the two cities, respectively. The results showed that LTPA was positively associated with walking/cycling facilities and crime safety in both cities. LTPA was positively correlated with residential density, aesthetics, pedestrian/traffic safety in Wenzhou and negatively correlated with access to services in Hangzhou. The perceived scores of aesthetics (2.71 vs. 2.45) and pedestrian/traffic safety (2.11 vs. 1.71) in Hangzhou were significantly higher than those in Wenzhou. The results suggested that built environment elements like higher walking/cycling facilities and crime safety may motivate elderly engaging LTPA in both cities. However, LTPA was affected by different factors in these two cities. In the urban redevelopment, survey conducted in its own city would provide meaningful information and cannot be neglected.

## 1. Introduction

According to the “National Assessment of Aging and Health in China” published by the World Health Organization (WHO) in 2016, China is undergoing a faster population aging process than most other countries in the world. In the future 25 years, the percentage of elderly aging over 60 years in the total population will be doubled, from 12.4% in 2010 to 24% in 2040. The number of elderly aging over 80 years will be 90.4 million in 2050 [1]. The health of elderly and successful ageing has become an important social topic in China. Physical activity has been suggested to be beneficial for both physical and mental health [2,3,4,5]. Epidemiological studies have presented that physical activity is associated with reduced risks of obesity, diabetes, cardiovascular disease, and other chronic diseases [6,7,8]. For elderly, moderate but regular physical activity has been shown to be associated with a reduction in total mortality among elderly by a review study. The benefits include a positive effect on prevention of coronary heart disease, stroke, type 2 diabetes, and so on [9]. Multiple types of physical activities have been recommended for elderly [9]. On the other hand, WHO suggested that LTPA could promote the elderly’s health the most due to the greatest energy consumption compared to the other three types of physical activities [10]. Therefore, increasing LTPA is critical for elderly health.

Elderly LTPA would be motivated by a satisfying physical and social environment. Previous studies have shown that the social support networks with friends and family members were positively related with elderly LTPA [11,12,13]. Boehm et al. found that the elderly with company of family or friends to walk had a 2.45 times higher prevalence of reaching LTPA recommendations than who did not [12]. There were some barriers for elderly engaging in LTPA, such as health status, fear, and weather [14]. Poor health was reported as a barrier for elderly physical activity [15,16]. Fear form different origins was another reported barrier for elderly physical activity, including fear for exercising outside, fear for injury, fear for falling, and so on [15]. There is also some evidence showing that rainfall has the largest negative correlation with elderly physical activity [17]. On the other hand, WHO suggested that age-friendly city topic areas include outdoor spaces and building, transportation, housing, social participation, respect and social inclusion, civic participation and employment, communication and information, community support and health services. All those factors improved might encourage elderly to take more outdoor physical activity and improve their health status [18].

There are vast scientific evidences from different countries showing that built environment is an important factor affecting physical activity [19,20,21,22,23,24]. However, the associations between built environment and physical activity level were still controversial because of different survey participants and places. Sun et al. found that residential density was negatively associated with women physical activity in China [25]. The similar results were also found in other Chinese studies [26,27]. However, a positive association was suggested in other countries [28,29,30,31]. The reason for controversial results may be due to the fact that the residential density in china was much larger than most countries in the world. A high residential density might make residents feel uncomfortable in their outdoor activities and unpleasant to engage in LTPA. Sun et al. also suggested that larger residential density leads to heavier traffic, and people in the denser city tend to choose shop online to avoid the traffic. This reduced the physical activity of people indirectly [25]. 

The relative studies were conducted in cities located in the east region [27,32,33], south region [34] and northwest region [25] of mainland, China. Most of these studies focused on adults over 18 years old [25,27,33,34], and only few studies aimed at the elderly over 60 years old [32]. Wu et al. found that street connectivity was positively associated with elderly physical activity in Nanjing [32]. However, a negatively association between street connectivity and physical activity of adults was suggested in Shanghai and Xi’an [25,33]. The different results were mainly caused by diverse participants. Elderly have much free time for LTPA. A good street connectivity provides elderly with alternative transportation routes to promote walking [32]. However, adults do not have so much free time to engage in LTPA. They may avoid traveling during rush hour, which reduces LTPA [25]. Some studies from Hongkong also focused on elderly [26,35], but the demographic profile and social-economic status of participants in Hongkong are slightly different from those conducted in mainland. This might affect the relationship between built environment and physical activity. Therefore, more elderly studies are needed to figure out the effect of built environment on elderly LTPA in mainland of China. 

To our knowledge, no study compared elderly physical activity and built environment of two different cities. Using a comparison method might provide a new insight into this field study, especially considering the variations in demographic profile and social-economic status of population in different cities. Therefore, the purpose of this study was to locate the association between built environment and elderly LTPA in a first-tier city Hangzhou and a second-tier city Wenzhou in the east region of China. Meanwhile, we would like to find the differences in elderly LTPA and perceived built environment scores of two cities. Finally, to discuss how the built environment affect elderly LTPA. 

## 2. Materials and Methods 

### 2.1. Sample and Study Design

This study was conducted in the cities of Hangzhou and Wenzhou from July to December in 2019. According to city classification published by China Business Weekly magazine in 2019, Hangzhou is a first-tier city and Wenzhou is a second-tier city mainly based on their economic development and future development potential. Both of them situate in Zhejiang province, the southeast coastal area of China. Hangzhou is famous for its beautiful environment. West Lake is located in the downtown of Hangzhou, and is free for everyone to visit. With five subway lines in Hangzhou and only one line in Wenzhou, public transportation is more advanced and convenient in Hangzhou. As the capital of Zhejiang Province, Hangzhou has hosted and will host quite a few global events, such as G20 Summit in 2016 and the Asian Games in 2022. Therefore, Hangzhou might be better in urban construction and service provision. Besides, Hangzhou is larger in both economy and population scales than Wenzhou. Hangzhou ranked at 9th in 2019 in terms of economic development in China, while Wenzhou ranked at 30th in 2019. By the end of 2019, the official resident population of Hangzhou reached 10,360,000, and the population of Wenzhou was 9,300,000. Additionally, the migrant population (having a permanent address outside of the city) in Hangzhou was larger than that in Wenzhou. The percentages of migrant population in total resident population were 43.4% and 31.9% in Hangzhou and Wenzhou, respectively.

In this study, all the selected districts were located in the downtown area based on the geographical location characteristics of the two cities. Five districts of Hangzhou were involved, including Shangchen, Xiacheng, Jianggan, Xihu and Gongshu districts. Two districts of Wenzhou were involved, including Lucheng and Ouhai Distircts. A cross-sectional survey of random samples of elderly was conducted in communities. 308 elderly from 10 different communities in Hangzhou and 304 elderly from nine different communities in Wenzhou were recruited with the help of community resident committees for this study. The inclusion criteria of participants were (a) elderly aging over 60 years, (b) be residents of the selected communities, (c) have lived in the area for at least 6 months, (d) have no cognitive impairment and with normal communication abilities. All participants involved in this study have signed written consents to participate. The study was approved by the ethics committee of Research Academy of Grand Health, Ningbo University (RAGH20190600021; June 2019).

### 2.2. Measures

Three questionnaires are involved in this study, including individual characteristics questionnaire, the Chinese version of Neighborhood Environment Walkability Scale—Abbreviated (NEWS-A) and International Physical Activity Questionnaire—short version (IPAQ-S). Individual characteristics questionnaire was used to collect data including sex, age, education level, income situation, travel mode choice, occupational status and motion sickness. The Chinese version of NEWS-A was modified based on NEWS-A developed in the USA for adults by a Hongkong research team. The reliability and validity of the modified questionnaire has been testified [36] and been used for Chinese elderly study [26,35]. The perceived scores of built environments were measured by the modified questionnaire in this study. IPAQ-S was used to collect elderly LTPA level during the past seven days. The IPAQ data were then converted into metabolic equivalent (MET) scores following the IPAQ scoring procedure. All the questionnaires were filled by our research team members through one-to-one interviews to ensure the quality. 

Eight elements were evaluated in the NEWS-A, including residential density, land use mix diversity, access to services, street connectivity, walking and cycling facilities, aesthetics, traffic safety, and crime safety. In the residential density element, the residential density and building type were assessed. Each item is measured on a 5-point Likert Scale (1-none, 5-all). For example, question 1: how common are detached single-family residences in your immediate neighborhood? (1-none, 2-a few, 3-some, 4-most, 5-all). Then the residential density was computed on the basis of formula suggested by a previous study [36]. In the land use mix diversity element, each item is measured on a 5-point Likert Scale (1-1 to 5 min, 5-more than 30 min). A higher score means a farther distance from home to destination. In the other six elements, each item is measured on a 4-point Likert Scale (1-totally disagree, 4-totally agree). Access to services included six questions and assessed the perceived convenience to exercise facilities (e.g., park), commercial facilities (e.g., supermarkets), public transportation stations (e.g., bus, subway) and road flatness. Street connectivity included three questions and assessed the number of dead-end streets, the distance between intersections, and the alternative routes. A higher score means a better street connectivity. Walking and cycling facilities consisted of six questions and assessed the quality and safety of sidewalks. Aesthetics included five questions and assessed neighborhood greening, cleanliness, cultural landscape, and attractive building. Traffic safety consisted of three questions and assessed traffic volume and safety. Crime safety consisted of three questions and assessed the crime rate, perceived walking safety in the day and at night. The final scores for those seven elements are the total score divided by the number of items in the corresponding element. A higher score indicates better perceived built environment. 

### 2.3. Statistical Analysis

Descriptive statistics frequency and percentage were used to describe sample demographics characteristics. Independent T-test was performed to examine the differences of elderly LTPA and perceived scores of built environments between Hangzhou and Wenzhou. Means and standard deviations were used to describe the built environment and elderly LTPA characteristics. A multivariate linear regression method with a univariate model was used to analyze the relationship between elderly LTPA and built environment elements in both cities. Statistical significance was set at *p* < 0.05. All the analyses were conducted by SPSS 19.0 software (SPSS Inc., Chicago, IL, USA). 

## 3. Results

Table 1 and Table 2 present the characteristics of the participants. In both cities, there was a larger number of females than males. Besides, the elderly who are 70–79 years old are the majority (40.9% in Hangzhou and 40.5% in Wenzhou) in the recruited participants. The education level of participants was slightly higher in Hangzhou compared to Wenzhou. In Hangzhou, the educational level of 66.6% of the participants was lower secondary or upper secondary, while in Wenzhou, 75% of participants were lower secondary or primary or below in Wenzhou. The income situation was also better in Hangzhou. 81.1% of elderly income were above 3501 Renminbi (RMB, Chinese currency) in Hangzhou. However, only 52% of elderly income were above 3501 RMB in Wenzhou. The main travel mode was car or bus in both cities. Concerning the other two travel modes, more elderly chose walking instead of bicycle in Wenzhou. 44.7% of elderly chose walking in Wenzhou compared with 22.4% of elderly in Hangzhou. Most of elderly were unemployed or retired in both cities. 70.5% and 57.2% of elderly were free of motion sickness in Hangzhou and Wenzhou, respectively. 

The MET values of elderly LTPA in both cities are shown in Table 3. The elderly LTPA in Wenzhou was significantly higher than that in Hangzhou (*p* < 0.001). The perceived scores of built environments in both cities are shown in Table 4. Except pedestrian/traffic safety and crime safety, the scores of the other six built environment elements were all above the median scores according to the ranges of the scores. Significant differences were found in residential density, street connectivity, aesthetics, pedestrian/traffic safety and crime safety between the two cities. The perceived scores of residential density, aesthetics, pedestrian/traffic safety and crime safety in Hangzhou were significantly higher than that in Wenzhou (*p* < 0.001). The perceived scores of street connectivity in Hangzhou was significantly lower than that in Wenzhou (*p* < 0.001). No significant differences were found in access to services, walking/cycling facilities and land use mix diversity. The association between elderly LTPA and built environment are shown in Table 5. The results showed that elderly LTPA in Wenzhou was positively associated with residential density, walking/cycling facilities, aesthetics, pedestrian/traffic safety and crime safety. The elderly LTPA in Hangzhou was positively associated with walking/cycling facilities, crime safety, and negatively associated with access to services.

## 4. Discussion

The purpose of this study was to identify any associations between built environment and elderly LTPA in a first-tier city Hangzhou and a second-tier city Wenzhou in the east region of China. On the other hand, we would like to find the differences of elderly LTPA and perceived built environment scores between the two cities. Finally, to investigate how the built environment elements influence elderly LTPA.

Our study implied that good walking/cycling facilities would motivate elderly to engage in LTPA. The results of this study showed that the perceived score of walking/cycling facilities was positively associated with elderly LTPA level in both cities. This finding was in line with previous studies [25,37,38]. Sun et al. found that walking/cycling facilities was positively associated with men LTPA in Xi’an, a northwest city of China. They suggested that good walking/cycling facilities provide new choices to travel faster in rush hour for residents [25]. For elderly, their bodily sensation and balance reduced with age increasing, and they usually are often particularly sensitive to physical barriers in the neighborhood environment. A bad walking/cycling facility might result in elderly a panic mentality, and hinder their engaging in LTPA. In contrast, a satisfying walking/cycling facility would motive elderly take more outdoor exercise [39]. The perceived scores of walking/cycling facilities were similar and no significant differences were found between Hangzhou and Wenzhou. Therefore, walking/cycling facilities might not be the cause of differences in elderly LTPA of the two cities.

Crime safety was another common built environment affecter of elderly LTPA in both cities. Our results showed that perceived scores of crime safety were positively associated with elderly LTPA. The social environment is an important factor and is likely to influence physical activity in elderly [40]. lower criminal activity will increase the chance of elderly going out to exercises, so improving their LTPA. The similar finding was also suggested in Hongkong elderly. Cerin et al. found that the odds of non-participation in LTPA was related with safety aspect of the neighborhood [26]. The finding in this study, in combination with previous literature findings highlights the linkage between social neighborhood attributes and elderly LTPA. However, other Chinese studies did not find an association between crime safety and resident LTPA in adults [27] and elderly [32]. According to the latest poll report published by the American company Gallup in 2020, China ranked third in law and order index [41]. Therefore, whether crime safety is still an important element influencing elderly LTPA in China needs more studies. Surprising, the T test results showed that the crime safety score in Wenzhou was significantly lower that it was in Hangzhou, but the elderly LTPA level in Wenzhou was significantly higher than that in Hangzhou. This result was a little in conflict with a positive association between crime safety and elderly LTPA in both cities. The conflict results might be explained by that the elderly LTPA was affected by various built environment elements, not only by crime safety. The crime safety differences did not explain the elderly LTPA diversities just by itself.

The perceived score of pedestrian/traffic safety was only positively associated with elderly LTPA in Wenzhou. A significant difference was also found in pedestrian/traffic safety between the two cities. The perceived score in Hangzhou was higher than it was in Wenzhou. The above results might indicate that in a good pedestrian/traffic safety environment, the traffic safety is not a main problem when elderly go out to take exercise. However, if the pedestrian/traffic safety environment is not good enough, the traffic safety become a problem worth of attention to ensure themselves safety for elderly. A better pedestrian/traffic safety would motive elderly to engage more in LTPA. A positive association between pedestrian/traffic safety and LTPA was also suggested in elderly [40,42] and adults [43,44] by previous studies. They suggested that Good pedestrian/traffic safety would make elderly feel safe in their outdoor activities and encourage them to engage more in LTPA. The positive association between the perception of pedestrian/traffic safety and elderly LTPA may suggest the need to advocate for policy and regulatory actions aimed at improving pedestrian/traffic safety, especially in Wenzhou.

The perceived score of aesthetics was also positively associated with elderly LTPA only in Wenzhou. Moreover, the aesthetics score in Wenzhou was significantly lower than it was in Hangzhou. These results suggested that aesthetics is not an affecter of elderly LTPA in a good aesthetics environment, but it becomes an affecter in a not so good aesthetics environment. The explanation of this result might be that in a good aesthetics environment, residents have already been familiar with such an environment and no attention will be paid on it. The other built environment elements like walking/cycling facilities, crime safety would be main issues of interest for elderly, such as in Hangzhou. Therefore, aesthetics would not affect elderly’s decision whether they go out for exercise. If the aesthetics is not good enough as elderly expect, it would affect their decisions of engaging in LTPA. A better aesthetics would promote elderly to take part in LTPA more. The finding in Wenzhou was consistent with previous papers [25,34,45,46,47]. They found that a good aesthetics would motivate residents to engage in more LTPA in central-northwest city Xi’an and southern city Shenzhen. They suggested that a pleasing and beautiful environment is friendly for residents to engage in LTPA, such as walking, cycling or running.

Residential density was positively associated with elderly LTPA in Wenzhou, but the association relationship was not found in Hangzhou. The result in Wenzhou was consistent with previous studies in which a positive relationship between residential density and LTPA was suggested in other countries [28,29,30,31] and in China [33], but not in line with other studies in China [25,26,27]. Zhou et al. found that residents from downtown areas were more positively associated with leisure-time PA than those living in the suburbs. Residential density was a significant positive predictor of recreational or leisure-based PA in Shanghai [33]. However, Su et al. suggested that lower values of residential density were associated with more time spent leisure-time walking by women in Hangzhou [27]. Although a negative association was also found in Hangzhou in our study, but the association was not significant. The reason for discrepancy of association between residential density and LTPA from diverse studies might be different participants of studies and social-economic status differences of population from diverse cities. Residential density of Wenzhou was significantly lower than that in Hangzhou. This result could be explained by different population and city scales between the two cities. 

Access to services was negatively associated with elderly LTPA in Hangzhou. This finding was not in line with previous studies [48,49]. Van et al. suggested that perceived short distances to services was significantly positively related to elderly walking/cycling behaviors in Belgium [49]. The reason for the discrepancy might be that distance to services in China is not so far away from the elderly’s homes. The perceived score of access to services was 2.94 in Hangzhou, which means the participants somewhat agreed with that access to services is within easy walking distance of their home according to a 4-point Likert Scale. Additionally, most of elderly are retired and have enough free time to walk to services. Therefore, a little farther but within walking distance to services might encourage elderly walk more, and in hence increase their LTPA.

In summary, although the significant differences in some built environment elements and elderly LTPA level between the two cities were found in this study, but it did not indicate those built environment elements would affect elderly LTPA. The elderly LTPA of one city was affected by its own built environment. The association between elderly LTPA and built environment should be established based on data collected from one city. On the other hand, the comparison results between the two cities provide more information about how the built environment affect elderly LTPA. A built environment would become an influencer of elderly LTPA when it is not so good as elderly expected, such as pedestrian/traffic safety and aesthetics in this study. 

The results of this study need to be interpreted in light of some limitations. First, a comparison method was used in order to locate the differences in elderly LTPA and perceived built environment scores between two cities in the present study. Although those results did not investigate the association of elderly LTPA with built environment directly, it can help understand how built environment affect elderly LTPA to some extent. The present study was the first time to compare built environment of first-tier and second-tier cities in China and may provide a new insight into built environment studies. Secondly, although intentionally recognized questionnaires of NEWS-A and IPAQ-S and one-to-one interviews were used in the present study, the self-reported measurement tools may introduce some measurement errors and self-report bias inevitably. Third, demographic characteristics, social and psychological factors were not combined to explain the effect of built environment on elderly LTPA in this study. Previous studies have shown that social support and self-efficacy may motivate resident’s PA [12]. The strength of this study is that two cities of different city scales in eastern China were included. Through a comparison of built environment and elderly LTPA in the two cities, how the built environment affect elderly LTPA might be better understood. The results in the current study adds new and scarce data to the evidence base of built environment and elderly LTPA studies.

## 5. Conclusions

Built environment elements like higher walking/cycling facilities and crime safety may motivate elderly engaging LTPA in both cities. Additionally, higher aesthetics, pedestrian/traffic safety and residential density in Wenzhou and lower access to services in Hangzhou would also promote elderly to engage in LTPA. A built environment would become an affecter of elderly LTPA when it is not so good as elderly expected, such as pedestrian/traffic safety and aesthetics in this study. The related sectors like urban construction department should take into consideration of some important built environment elements in the urban redevelopment and construction process in order to develop an environment that motivate elderly LTPA. More studies on elderly LTPA and perceived built environment are needed in the future to provide information of the association differences at city level.

## Figures and Tables

**Table 1:** Demographics of the participants in Hangzhou (*n =* 308).

Demographic Variable	*n*	*%*
Gender		
Men	116	37.7
Women	192	62.3
Age		
60–69 years	117	38.0
70–79 years	126	40.9
≥80 years	65	21.1
Education level		
Primary or below	50	16.2
Lower secondary	117	38
Upper secondary	88	28.6
Tertiary and above	53	17.2
Income situation (RMB)		
≤1500	6	1.9
1501–2500	9	2.9
2501–3500	43	14.0
3501–4500	118	38.3
≥4501	132	42.9
Travel mode choice		
Car or bus	178	57.8
Bicycle	61	19.8
Walking	69	22.4
Motion sickness		
Yes	91	29.5
No	217	70.5

Note: Income situation indicates the monthly income of the participant. % represents the percentage of each variable in the total participants in Hangzhou. RMB stands for the Chinese currency.

**Table 2:** Demographics of the participants in Wenzhou (*n =* 304).

Demographic Variable	*n*	*%*
Gender		
Men	121	39.8
Women	183	60.2
Age		
60–69 years	81	26.6
70–79 years	123	40.5
≥80 years	100	32.9
Education level		
Primary or below	122	40.1
Lower secondary	106	34.9
Upper secondary	49	16.1
Tertiary and above	27	8.9
Income situation (RMB)		
≤1500	41	13.5
1501–2500	18	5.9
2501–3500	87	28.6
3501–4500	85	28.0
≥4501	73	24
Travel mode choice		
Car or bus	141	46.4
Bicycle	27	8.9
Walking	136	44.7
Motion sickness		
Yes	130	42.8
No	174	57.2

Note: Income situation indicates the monthly income of the participant. % represents the percentage of each variable in the total participants in Wenzhou. RMB stands for the Chinese currency.

**Table 3:** Comparison of elderly LTPA between Hangzhou and Wenzhou.

Variable	Hangzhou	Wenzhou	*t*	*p*
LTPA (MET.min/week)	2048.1 ± 1886.6	2676.7 ± 2386.9	−3.617	<0.001 *

Note: MET represents metabolic equivalent score, * indicates significant differences between Hangzhou and Wenzhou (*p* < 0.05).

**Table 4:** Comparison of perceived built environment scores between Hangzhou and Wenzhou.

Variable	Hangzhou	Wenzhou	*t*	*p*
Residential density	674.30 ± 180.40	599.10 ± 68.80	6.817	<0.001 *
Access to services	2.94 ± 0.49	2.88 ± 0.33	1.645	0.101
Street connectivity	3.16 ± 0.71	3.37 ± 0.48	−4.447	<0.001 *
Walking/cycling facilities	3.10 ± 0.39	3.09 ± 0.38	0.431	0.667
Aesthetics	2.71 ± 0.57	2.45 ± 0.40	6.437	<0.001 *
Pedestrian/traffic safety	2.11 ± 0.84	1.71 ± 0.62	6.633	<0.001 *
Crime safety	1.63 ± 0.81	1.32 ± 0.45	5.735	<0.001 *
Land use mix diversity	2.74 ± 1.02	2.73 ± 0.55	0.188	0.851

Note: * indicates significant differences between Hangzhou and Wenzhou (*p* < 0.05).

**Table 5:** Association between built environment and LTPA in Hangzhou and Wenzhou.

Variable	B	Hangzhou SE	*p*	B	Wenzhou SE	*p*
Residential density	−0.96	0.58	0.10	4.24	1.86	0.03 *
Access to services	−789.51	228.74	0.001 *	−486.85	405.90	0.23
Street connectivity	97.45	162.39	0.55	−36.57	269.64	0.89
Walking/cycling facilities	1016.69	236.44	<0.001 *	1130.47	347.56	0.001 *
Aesthetics	28.72	193.25	0.88	863.18	332.04	0.01 *
Pedestrian/traffic safety	256.46	150.43	0.09	477.58	219.17	0.03 *
Crime safety	520.49	161.05	0.001 *	1740.41	294.04	<0.001 *
Land use mix diversity	27.60	101.217	0.79	−201.36	239.80	0.40

Note: Depend variable: total score of elderly LTPA, B is the regression coefficient, SE is the stand error, * indicates significance (*p* < 0.05).
